# *Angelica sinensis* Suppresses Body Weight Gain and Alters Expression of the* FTO* Gene in High-Fat-Diet Induced Obese Mice

**DOI:** 10.1155/2017/6280972

**Published:** 2017-09-20

**Authors:** Tao Zhong, Xiao-Yue Duan, Hao Zhang, Li Li, Hong-Ping Zhang, Lili Niu

**Affiliations:** Farm Animal Genetic Resources Exploration and Innovation Key Laboratory of Sichuan Province, College of Animal Science and Technology, Sichuan Agricultural University, Chengdu 611130, China

## Abstract

The root of* Angelica sinensis* (RAS) is a traditional Chinese medicine used for preventing and treating various diseases. In this study, we assessed RAS supplementation effects on body weight and the* FTO* gene expression and methylation status in a high-fat-diet (HFD) induced obese mouse model. Female obese mice were divided into groups according to RAS dosage in diet as follows: normal diet, HFD diet (HC), HFD with low-dosage RAS (DL), HFD with medium-dosage RAS (DM), and HFD with high-dosage RAS (DH). After RAS supplementation for 4 weeks, body weight suppression and* FTO* expression in DH mice were significantly higher than in HC mice, whereas no significant change in* FTO* expression was detected between DM and DL mice or in their offspring. Bisulfite sequencing PCR (BSP) revealed that the CpG island in the* FTO* promoter was hypermethylated up to 95.44% in the HC group, 91.67% in the DH group, and 90.00% in the normal diet group. Histological examination showed that adipocytes in the DH group were smaller than those in the HC group, indicating a potential role of RAS in obesity. This study indicated that RAS could ameliorate obesity induced by HFD and that the molecular mechanism might be associated with the expression of the* FTO* gene.

## 1. Introduction

Obesity is a global health priority and resistance to diet induced obesity has been studied in many animal models. A diet rich in sugar and containing up to 45% fat has been widely used to induce obesity in mice [1–3]. Scientists have found that some Chinese herbs like berberine, curcuma longa, and* Sibiraea angustata* could effectively ameliorate obesity by inhibiting the synthesis, growth, and accumulation of fatty acid in adipocytes [4–6]. The functions of Chinese herbs have been investigated* in vivo *in mouse models of high-fat-diet (HFD) induced obesity [7].

The root of* Angelica sinensis* (Chinese named Danggui), a well-known herbal medicine, has been historically used as a tranquilizer or a tonic agent [8, 9]. As a functional food, RAS can also be used for the amelioration of inflammation, diabetes, and cardiovascular disorders. Previous studies have shown that the* Angelica sinensis* polysaccharide (ASP), the active chemical components of RAS, had hematopoietic effects in animal and cellular models [10]. Two acidic polysaccharides (APS-3b and APS-3c) significantly inhibited the growth of S180 tumors and increased the life span of S180 tumor-bearing mice [11]. Furthermore, the antioxidant effects of ASP could stimulate the endothelial production of nitric oxide and resist ischemia/reperfusion (I/R) injury [12].

The fat mass and obesity associated* (FTO)* gene has a relative effect on obesity [13].* FTO* is located on chromosome 16 in humans and encodes a protein with a double-stranded b-helix fold, homologous to the members of the nonheme and 2-oxoglutarate oxygenase superfamily (which mainly impact the metabolism of fatty acid) [14]. Further, studies in humans and rodents suggested that* FTO *is involved in food intake regulation and lipid metabolism [15]. Histological studies revealed that the localization of* FTO* mRNA and protein in the hypothalamic nucleus was of critical importance in the regulation of feed intake in mice [16]. Loss of function of* FTO* in mice led to postnatal growth retardation and a significant reduction in adipose tissue and lean body mass [17]. Over expression of* FTO*, however, led to a dose-dependent increase in body and fat mass [18]. Furthermore, Merkestein and colleagues found that* FTO *influences adipogenesis by regulating the process of mitotic clonal expansion in obese mice [19].

In the present study, we assessed the effects of RAS supplementation on body weight in HFD mice. Furthermore, mRNA expression and promoter methylation status of the* FTO* gene were determined to compare the effects of RAS between control and treated groups. This study will provide a foundation for understanding the functions of RAS in obesity resistance.

## 2. Materials and Methods

### 2.1. Ethics Statement

Animas involved in this study were sacrificed according to the Regulations for the Administration of Affairs Concerning Experimental Animals and approved by the Institutional Animal Care and Use Committee at the College of Animal Science and Technology, Sichuan Agricultural University, Sichuan, China, under the permit number DKY-B20110807.

### 2.2. Animals and Diets

Female KM mice at 5 weeks of age were obtained from the Chengdu Dossy Laboratory Animal Co., Ltd, in Sichuan province, China. Mice were reared at standard conditions within a free access to food and water for 1 week to acclimatize to experimental conditions. Mice were then randomly assigned to the normal diet group (GC, *n* = 10) or the HFD group (HC, *n* = 48) without RAS supplementation. After 5 weeks, the obese mice were randomly divided into four groups. All groups were continually fed with HFD. The first group (*n* = 12) was designated the high-fat control group (HC) without RAS supplementation. The second group (*n* = 11) received RAS supplementation at 2.00 g/kg·BW and was designated as the low-dose group (DL). The third group (*n* = 11) received RAS supplementation at 5.00 g/kg·BW and was designated the medium-dose group (DM) and the last group (*n* = 12) was designated the high-dose group (DH) with RAS supplementation at 10.00 g/kg·BW. The female offspring were obtained by mating with the male KM fed under same conditions for each group. The diet formula is shown in Table 1. The RAS was dried and ground and then added to the mixture, which was kneaded and made into a cylindrical shape using 5-mL injectors. Finally, the mixture was dried at 60°C overnight and stored in a hermetic bag after cooling to room temperature. Body weight was measured weekly. Adipose tissues were collected from 4 mice in each group after RAS supplementation for 35, 60, and 95 days.

### 2.3. Total RNA Extraction and Reverse Transcription

Mice were sacrificed by cervical dislocation and adipose tissue was collected and rapidly frozen in liquid nitrogen and then stored at −80°C. Total RNA was extracted using TRIzol reagent (Invitrogen, CA, USA). The purity of the isolated RNA was determined by agarose gel electrophoresis and the concentration was quantified by the ND-2000 Nanodrop (Thermo Scientific, MA, USA). cDNA was synthesized using the Prime Script RT reagent Kit (Takara, Dalian, China) according to the manufacturer's recommendations.

### 2.4. Quantitative Real-Time PCR of the* FTO* Gene

We performed quantitative real-time PCR (qPCR) to quantify the relative mRNA expression levels of* FTO* in adipose tissues of mice from GC, HC, DL, DM, and DH groups, as well as DH offspring. Primer pairs used for qPCR (Table 2) were designed according to the mouse* FTO* gene (NM_011936). The qPCR was performed in triplicate using a SYBR® Premix Ex Taq™ II kit (Takara, Dalian, China). Each qPCR reaction (total volume 10 *μ*L) contained 0.8 *μ*L cDNA, 5 *μ*L SYBR Green II, 0.8 *μ*L primer pairs, and 3.4 *μ*L ddH_2_O. The qPCR procedures were as follows: 95°C for 3 min, 40 cycles of 95°C for 10 s, 30 s at optimum temperatures, 72°C for 10 s, and a final extension for 5 min, and then a temperature increment of 0.5°C/s from 65°C to 95°C to build a melting curve. The specificity of qPCR products was confirmed by melting curve analysis. The relative expression of* FTO *mRNA was determined using the geometric mean of* GAPDH, β-actin, *and* 18s rRNA *by the 2^−ΔΔCT^ method [20].

### 2.5. DNA Preparation and Methylation Analysis by BSP Method

Genomic DNA was extracted from the adipose tissues of mice from GC, HC, and DH groups after RAS supplementation for 35 days, using TIANamp Genomic DNA Kit (Tiangen, Beijing, China). Three animals were randomly selected from each group. DNA was quantified by the ND-2000 Nanodrop and then treated with sodium bisulfite using the EZ DNA Methylation Gold Kit (Zymo Research, CA, USA). Following the manufacturer's instructions, DNA dosage was strictly limited to insure complete cytosine to uracil conversion.

The methylated CpGs in the* FTO* promoter (Acc. number AC105989) were estimated by the online program (http://www.urogene.org/cgi-bin/methprimer/methprimer.cgi). The primer pairs were modified by Primer Premier 5 (Table 2). The PCR was run on a C1000 PCR system Thermocycler (Bio-Rad, Richmond, CA) in a volume of 30 *μ*L including 1 *μ*L bisulfite-converted DNA or 0.5 *μ*L PCR products, 15 *μ*L Zymo Taq™ PreMix, or 1 *μ*L primer pairs. PCR reactions were as follows: 95°C for 5 min, 40 cycles of 30 s at 95°C, 30 s at 65.2°C (second round: 57.5°C), and 30 s at 72°C, and a final extension for 7 min at 72°C. The second PCR products were purified with a DNA Gel Extraction Kit (TsingKe, Chengdu, China) and then ligated into pMD 19-T vector (Takara, Dalian, China). At least ten positive recombinants were sequenced on an ABI 3730XL DNA analyzer (Applied Biosystems, USA). The methylated CpGs were analyzed with the QUantification tool (QUMA, http://quma.cdb.riken.jp/).

### 2.6. Histological and Morphometric Analysis

The abdominal adipose tissues were resected and fixed in 4% paraformaldehyde after washing. They were then dehydrated in ethanol and soaked in dimethylbenzene and then embedded in paraffin. The tissue blocks were sectioned at 4-micron thickness and then stained with haematoxylin and eosin (H&E) reagent. Photomicrographs were obtained and analyzed using Image-Pro Plus software (Media Cybernetics).

### 2.7. Statistical Analyses

All of the statistical analyses were performed using SPSS 20.0 (SPSS, Chicago, IL, USA) and the data were represented as means ± SD. The significance between groups was estimated by a one-way ANOVA test and Student's *t*-test.

## 3. Results

### 3.1. Effects of RAS Supplementation on Body Weight in HFD Fed Mice

**Figure 1:**
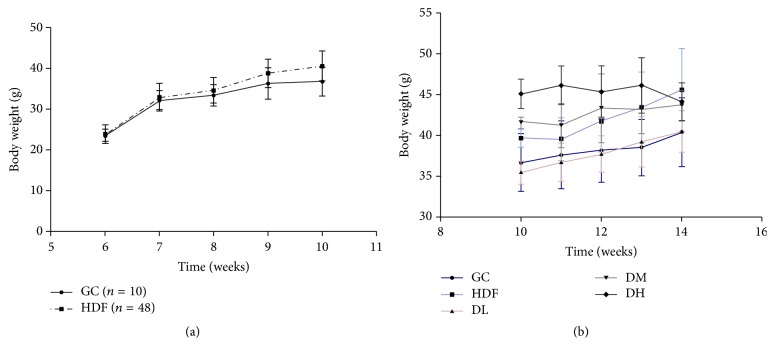
Body weight changes in the HFD mice model (a). Effects of RAS supplementation on body weight alterations in the HFD mice (b).

Compared with the normal diet group (GC), the HC mice showed marked obesity after feeding with HFD for 5 weeks (Figure 1(a)). During HFD treatment, appetite, activity, and coat luster were normal for all animals. After 5 weeks, the mice in the HC group were divided into five subgroups, HC, DL, DM, and DH, fed with different dosage of RAS supplementation for another 4 weeks. Compared with the mice in the GC, HC, DL, and DM groups, the mice in the DH group showed the lowest weight gain (Figure 1(b) and Table 3). We later used 10.00 g/kg·BW RAS to feed DH offspring continually. However, RAS supplementation did not show an obvious effect on body weight between the offspring from the HC and DH groups (data not shown).

### 3.2. Effects of RAS Supplementation on* FTO* mRNA Expression

**Figure 2:**
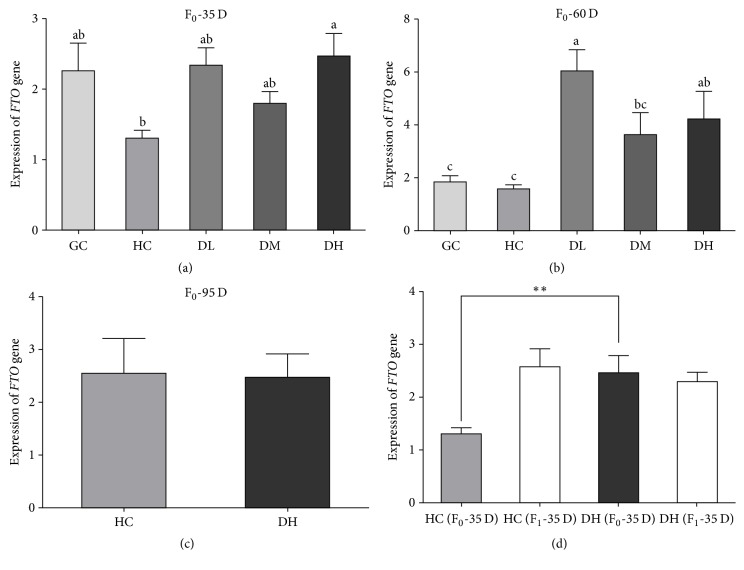
Effects of RAS supplementation on* FTO* mRNA expression in HFD obese mice. Different letters above each bar represent being significantly different (*P* < 0.05). *∗∗* indicates significant difference (*P* < 0.01).

To assess the effects of RAS supplementation on* FTO* mRNA expression, we performed qPCR to quantify* FTO* expression in adipose tissues collected from mice in the GC, HC, DL, DM, and DH groups at days 35 and 60. In addition, we also determined* FTO* expression in HC and DH mice at day 95, as well as in their progeny at day 35. The qPCR results showed that the expression of* FTO* in the RAS-supplemented groups (DL, DM, and DH) was significantly higher than in the HC group after both 35 d and 60 d (Figures 2(a) and 2(b)). No significant difference was detected between the HC and DH groups after 90 d (Figure 2(c)). Moreover, there was no significant difference in* FTO* expression between the offspring of mice in the HC and DH groups (Figure 2(d)).

### 3.3. Effects of RAS Supplementation on Methylation in the* FTO* Promoter

**Figure 3:**
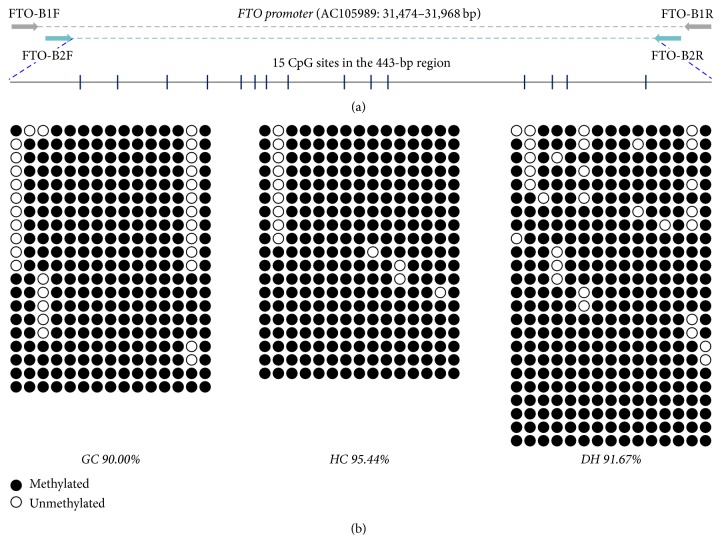
Schematic representation of the* FTO* promoter and the intragenic CpG sites in a 443-bp region (a). Methylation status of the* FTO* promoter in adipose tissues in GC, HC, and DH mice after being RAS-treated for 35 days (b). Each line represented as a sequence from each clone, while each vertical bar corresponded to an identical CpG site.

The structure of the analyzed CpG sites and their locations (GenBank Acc. number AC105989: 31474–31968) within the* FTO* gene are shown in Figure 3. The DNA methylation patterns of the GC, HC, and DH groups were assessed by bisulfite sequencing. The region analyzed includes a defined CpG island, located 666 bp upstream of the start codon. The* FTO* gene was hypermethylated in GC mice (90.00%), while the mice fed with HFD showed a higher methylation level in the HC group (95.44%). As expected, supplementation of RAS reduced the methylation level, which reached 91.67% in DH mice.

### 3.4. Effects of RAS Supplementation on Adipocyte Morphology

**Figure 4:**
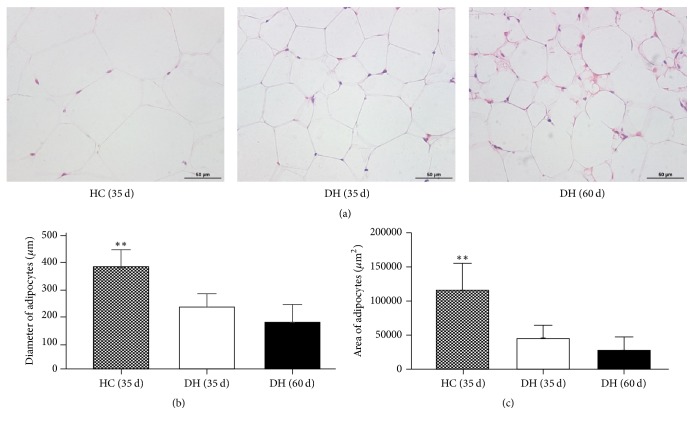
Histological assessment of the adipose tissues of mice in the HC and DH groups (a). Scale bars, 50 *μ*m. The quantitative data of the mean diameter and area of adipocytes are shown (b). Values are mean ± SD (*n* = 3). ^*∗∗*^*P* < 0.01.

The H&E histological examination showed that adipocytes had a polygonal morphology with typical peripherally located nuclei and distinct cell borders (Figure 4(a)). After being fed HFD with RAS supplementation for 35 d, the volume of adipocytes in the DH group was smaller than that in the HC group and showed some fibrous tissue in the intercellular substance and a greater number of adipocytes (Figure 4(a)). Adipocytes in DH mice fed RAS for 60 d were smaller again and the cell arrangement was obviously abnormal compared with HC mice. Histological and quantitative analyses revealed that the mean diameter of adipocytes in the HC and DH mice fed RAS for 35 d was 373.9 ± 64.8 *μ*m and 227.7 ± 48.2 *μ*m, respectively, and adipocyte area was 115,817.8 ± 38,956.0 *μ*m^2^ and 44,655.3 ± 19,589.2 *μ*m^2^, respectively (Figure 4(b)). In the DH mice fed RAS for 60 d, the mean diameter and area of adipocytes were 171.4 ± 66.0 *μ*m and 27,303.4 ± 20,430.6 *μ*m^2^, respectively. Overall, the mean diameter and area of adipocytes in HC mice were significantly greater than in DH mice (*P* < 0.05).

## 4. Discussion

In this report we demonstrate, for the first time, the favorable effects of RAS supplementation on body weight, gene expression, and promoter methylation of the* FTO* gene in mice with HFD obesity. As many previous studies, the obesity mouse model was induced by HFD; while formulas differ between studies, common ingredients included sugar and lard [1, 2]. The obesity mice model was established by HFD and the standard deviation both in GC and HC groups was accredited. Intragroup inconsistencies in body weight after the administration of RAS may have been due to individual error [8, 21]. In the present study, body weight gain was lowest in the DH group, which received 10.00 g/kg·BW of RAS supplementation (Table 3). Meanwhile, body weight gain in the mice fed with low or moderate dosage (DL and DM groups) was not different from the HC mice, indicating that a higher dosage of RAS supplementation (10.00 g/kg·BW) might be more beneficial in suppressing body weight in obese individuals.

We found that the expression of* FTO* mRNA was significantly increased in RAS-supplemented mice (DL, DM, and DH groups) and body weight gain of HC mice was markedly higher than that of DL, DM, and DH mice. This suggests that RAS might influence BW gain via alterations of gene expression. As showed by qPCR, there was a significant difference in* FTO* expression between RAS-supplemented groups and the GC and HC groups in 35 d, suggesting a correlation between* FTO* expression and body weight gain. In HC mice, however,* FTO *expression was lowest and obesity was highest. Previous studies reported that loss of* FTO *in mice resulted in a significant reduction of adipose tissue and lean body mass [17], and that* FTO* influenced adipogenesis by regulating events early in adipogenesis during the process of mitotic clonal expansion [19].* FTO* mRNA was more highly expressed in RAS-treated groups than the control group, especially the DH group. After 60 d, the highest* FTO* expression level was in the RAS-treated groups. Moreover, no significant difference was observed after 95 d between the GC and HC groups, or in their offspring. These results suggest that the effects of RAS supplementation on* FTO* expression are not heritable.

*FTO* is a nucleic acid demethylase that removes methyl groups from both DNA and RNA [16, 22]. Previous studies have shown that DNA methylation is altered not only in oocytes of obese mice but also in their offspring [23]. The modification of DNA methylation provides a link between the environment and gene expression, therefore, we investigated the methylation levels of CpGs in the* FTO* promoter region. There are 15 CpG dinucleotides present in the 2 Kb upstream of the start codon of* FTO*. BSP results showed that the CpG sites within the* FTO* promoter were highly methylated in all of the three groups (GC, HC, and DH). Although the methylation levels were not significantly different, our results revealed that promoter methylation and expression of* FTO* gene mRNA were negatively correlated. Thus, regulation by a methyl group blocking the transcription factor binding to this region (AC105989: 31,474–31,968 bp) is possible.

Previous study has demonstrated the polysaccharide of* Angelica sinensis* in ameliorating glucose and lipid metabolism disorders related to obesity, possibly due to interactions with insulin and serum inflammatory factors [8]. The molecular mechanisms, however, remained unclear. In the present study, the extent of the adipose tissues was different between HC and DH mice. We suggested that this difference in body weight may be caused by adipocyte morphology. Furthermore, we performed histological examination to view the alterations in adipocyte shape and size between HC and DH mice. As shown in Figure 4, adipocytes in the HC group were more mature and characterized by bigger lipid droplets, while those in the DH had more and smaller lipid droplets. The mice in the DH group fed RAS for 60 d showed much smaller adipocytes and more intercellular substance than DH mice fed RAS for 35 d.

## 5. Conclusion

This study investigated whether RAS could suppress body weight in HFD obese mice. The supplementation of RAS was associated with suppression of body weight, increased* FTO* mRNA expression, and reduction of methylation. The present study provides new insights into the biological role of RAS. Further detailed analyses need to be performed to understand the mechanism of RAS in body weight suppression.

## Figures and Tables

**Table 1:** Composition of the experimental diets used in the present study.

Ingredients	Normal diet	High-fat-diet			
	GC	HC	DL	DM	DH
Corn starch	30.0%	22.0%	21.6%	21.0%	19.6%
Flour	30.0%	22.0%	21.6%	21.0%	19.6%
Bran	14.0%	10.0%	10.0%	9.6%	9.0%
Soybean meal	20.0%	15.0%	14.8%	14.4%	13.4%
Fish meal	6.0%	6.0%	6.0%	5.6%	5.0%
Lard	0	8.0%	7.8%	7.6%	7.4%
Egg	0	10.0%	10.0%	9.6%	9.0%
Sucrose	0	7.0%	6.8%	6.6%	6.2%
*Angelica sinensis*	0	0	1.4%	4.6%	10.8%
Total	100.0%	100.0%	100.0%	100.0%	100.0%

**Table 2:** Information of primer pairs used in this study.

Primer name	Primer sequence (5′-3′)	Size (bp)	Tm (°C)
QPCR			
FTO-Q-1F	GAGTTCTATCAGCAGTGG	163	55.0
FTO-Q-1R	GCACATCTTTGCCTTGGA		
GAPDH-1F	GGTGAAGGTCGGTGTGAACG	233	55.0
GAPDH-1R	CTCGCTCCTGGAAGATGGTG		
ACTB-1F	CGTTGACATCCGTAAAGACC	281	55.0
ACTB-1R	AACAGTCCGCCTAGAAGCAC		
18S rRNA-1F	AGGGGAGAGCGGGTAAGAGA	241	55.0
18S rRNA-1R	GGACAGGACTAGGCGGAAC		
BPS			
FTO-B1F	TAGTTGATTTTGTTTGAAGAGGAAGA	671	65.2
FTO-B1R	TCCTACTCACTATCAACAATTCCTAA		
FTO-B2F	GGGTTGAAGAGGTGGTTTAGTAGTTA	496	57.5
FTO-B2R	ACAATCTCACTCAATCCACTTACATCT		

**Table 3:** Effects of RAS supplementation on body weight alterations in mice.

Group	AS content^*∗*^	Sample size	10 weeks	11 weeks	12 weeks	13 weeks	14 weeks
GC	NA	10	36.70 ± 3.52	37.62 ± 4.14	38.16 ± 3.90	38.50 ± 3.43	40.40 ± 4.20
HC	NA	12	39.71 ± 1.13	39.57 ± 2.62	41.75 ± 3.75	43.46 ± 4.32	45.60 ± 5.08
DL	2.00 g/kg·BW	11	35.50 ± 1.43	36.74 ± 2.32	37.72 ± 2.22	39.24 ± 3.09	40.50 ± 2.53
DM	5.00 g/kg·BW	11	41.60 ± 0.65	41.26 ± 2.71	43.34 ± 4.20	43.17 ± 2.92	43.70 ± 1.85
DH	10.00 g/kg·BW	12	45.10 ± 1.80	46.15 ± 2.37	45.38 ± 3.14	46.14 ± 3.37	44.20 ± 2.27

^*∗*^The dosage of AS was according to the recommendation.
